# Partition Function Zeros of the Spin-One Ising Model on the Honeycomb Lattice in the Complex Temperature Plane

**DOI:** 10.3390/e27121258

**Published:** 2025-12-15

**Authors:** Seung-Yeon Kim

**Affiliations:** School of Liberal Arts and Sciences, Korea National University of Transportation, Chungju 27469, Republic of Korea; sykimm@ut.ac.kr

**Keywords:** spin-one ising model, honeycomb lattice, partition function zeros

## Abstract

The spin-one Ising model on the honeycomb lattice has never been solved exactly in spite of its simplicity. Even its exact critical temperature is not known. The exact integer values for the density of states of the spin-one Ising model on the L×2L honeycomb lattice are enumerated up to L=14. The partition function zeros in the complex temperature plane of the spin-one Ising model on the L×2L honeycomb lattice are exactly obtained, using the density of states. The properties of the partition function zeros in the complex temperature plane are related to the behaviors of various thermodynamic functions, in particular, their singular behaviors. The unknown properties of the spin-one Ising model on the honeycomb lattice are investigated, based on its partition function zeros in the complex temperature plane.

## 1. Introduction

Phase transitions are among the most universal phenomena in the cosmos. The Ising model is a simple magnetic spin system on a lattice, introduced for understanding the occurrence of a ferromagnetic phase transition [1]. The one-dimensional Ising model in an external magnetic field was exactly solved, but it showed the paramagnetic–ferromagnetic phase transition only at zero temperature [1]. Later, Onsager found the exact solution of the spin-half Ising model on the square lattice in the absence of an external magnetic field, yielding the paramagnetic–ferromagnetic phase transition at a finite temperature and the logarithmic divergence of the specific heat at the transition temperature [2]. Following the Onsager solution, the exact solutions of the spin-half Ising model on various two-dimensional lattices have been obtained in the absence of an external magnetic field [3,4]. Since then, the Ising model has played a central role in our understanding of diverse phase transitions [5,6,7,8]. The Ising model has been the starting point for the modern theory of phase transitions and critical phenomena [6].

Yang and Lee introduced the idea of studying the zeros of the grand partition function in the complex fugacity (or magnetic field) plane to investigate the unsolved problem of the ferromagnetic Ising model in an external magnetic field [9,10]. They discovered the circle theorem that all Yang–Lee zeros of the ferromagnetic Ising model lie on the unit circle in the complex fugacity plane, independent of lattices and dimensions [10]. Furthermore, as the interactions are varied, the motion of the Yang–Lee zeros of the ferromagnetic Ising model has been studied [11,12]. Similarly, Fisher introduced the concept of the partition function zeros in the complex temperature plane of the spin-half Ising model on the square lattice [13]. Fisher showed that all Fisher zeros of the isotropic spin-half Ising model on the square lattice in the absence of an external magnetic field lie on two circles in the complex temperature plane, crossing the positive real axis at the ferromagnetic critical point and the antiferromagnetic critical point. Van Saarloos and Kurtze [14] showed that the partition function zeros in the complex temperature plane of the anisotropic spin-half Ising model cover domains and do not lie on curves. Because the partition function zeros of a given physical system provide the information on its exact properties, the theory of partition function zeros has been applied to diverse fields of physics from particle physics to biophysics [15,16,17,18,19,20,21,22,23,24].

The spin-one Ising model in two dimensions has never been solved exactly in spite of its simplicity. Even its exact critical temperature is not known in two dimensions. Warnaar et al. [25,26] found that the solvable restricted solid-on-solid dilute $A_{3}$ model is related to the two-dimensional critical spin-one Ising model in a magnetic field, and obtained the magnetic (or critical-isotherm) critical exponent δ=15 without the use of scaling relations. In this paper, we enumerate the exact integer values for the density of states of the spin-one Ising model on the L×2L honeycomb lattice up to L=14 (Section 2). Using the density of states, we evaluate the precise distributions of the partition function zeros in the complex temperature plane of the spin-one Ising model on the L×2L honeycomb lattices, and then we investigate the unknown properties of the spin-one Ising model on the honeycomb lattice (Section 4). In Section 3, we review briefly the partition function zeros in the complex temperature plane of the spin-half Ising model on the honeycomb lattice, whose properties are well known [27,28], to compare with those of the spin-one Ising model.

## 2. Density of States for the Spin-One Ising Model on the Honeycomb Lattice

The Hamiltonian of the spin-one Ising model on the honeycomb lattice with $N_{s}$ sites and $N_{b}$ bonds is defined by

(1) $$H=J\sum_{\langle i,j\rangle }(1-\sigma_{i}\sigma_{j}),$$

where a magnetic spin $\sigma_{i}$ can take values −1, 0, or +1 at each site *i*, *J* is the coupling constant between a nearest-neighbor spin pair *i* and *j*, and 〈i,j〉 is a sum over all nearest-neighbor pairs. If we define the density of states, Ω(E), with an energy index ($E=0,1,2,\ldots ,2N_{b}$)

(2) $$E=\sum_{\langle i,j\rangle }(1-\sigma_{i}\sigma_{j}),$$

the partition function $Z=\sum_{\left\{\sigma_{n}\right\}}e^{-\beta H}$ of the spin-one Ising model (a sum over all $3^{N_{s}}$ spin configurations) can be written as

(3) $$Z\left(y\right)=\sum_{E=0}^{2N_{b}}\Omega \left(E\right)y^{E},$$

where $\beta ={\left(k_{B}T\right)}^{-1}$ and $y=e^{-\beta J}$. The variable *y* is the so-called low-temperature variable [6], confined to the interval [0,1] for ferromagnetic interaction J>0.

**Figure 1:**
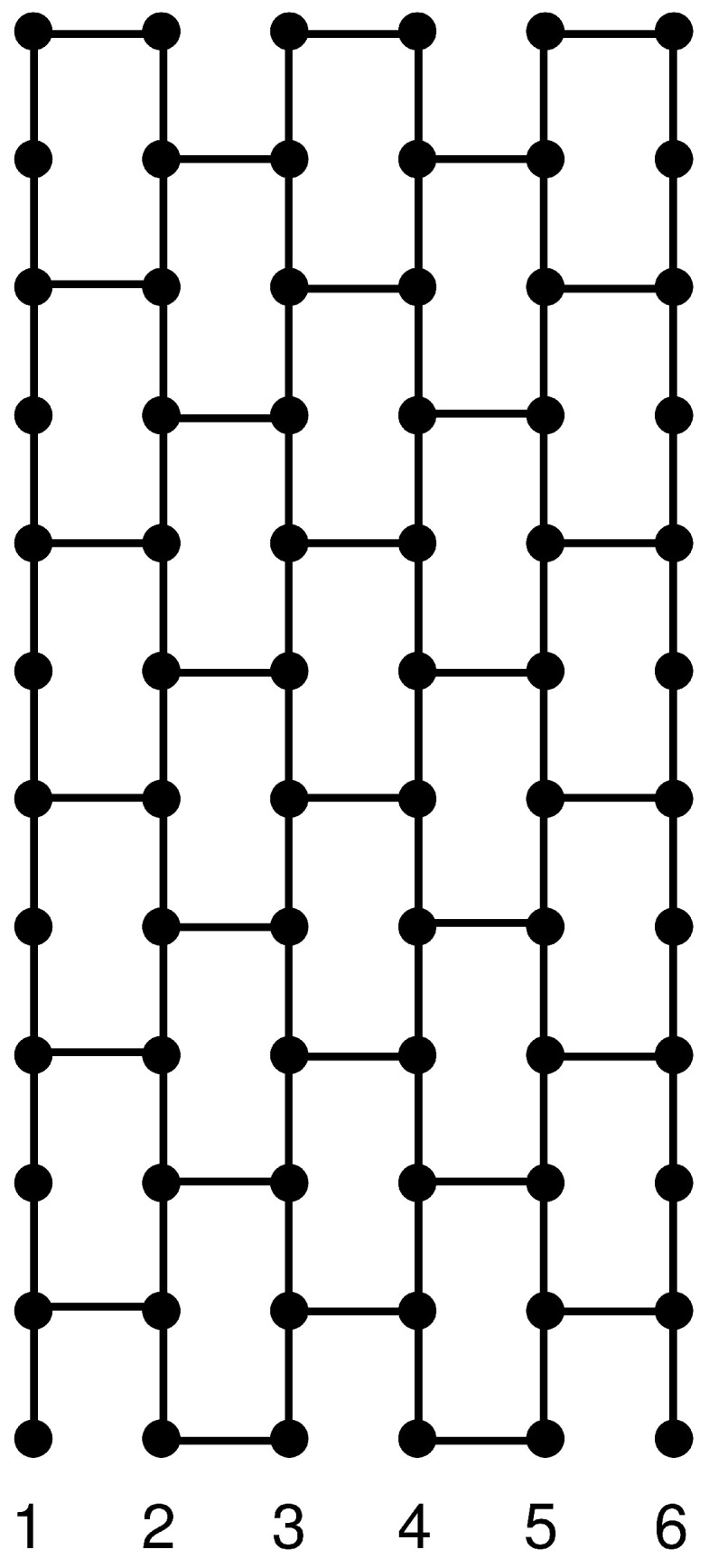
L×2L honeycomb lattice (L=6) with $N_{s}=2L^{2}=72$ sites and $N_{b}=3L^{2}-2L=96$ bonds.

The exact enumeration method for evaluating the density of states Ω(E) of the spin-half Ising model [29,30,31,32,33,34,35] is generalized to obtain the exact integer values for Ω(E) of the spin-one Ising model on the L×2L honeycomb lattice (up to L=14) with $N_{s}=2L^{2}$ sites and $N_{b}=3L^{2}-2L$ bonds, as shown in Figure 1. Table 1 shows the exact integer values for the density of states Ω(E) of the spin-one Ising model on the 5×10 honeycomb lattice with $N_{s}=50$ sites and $N_{b}=65$ bonds. For L=5, classifying all $3^{N_{s}}=3^{50}$ (≈7.179 ×$10^{23}$) states according to their energy values is an easy work. As shown in the table, the maximum density of states is Ω(65)=53720458126972059836125 (≈5.372 ×$10^{22}$). For L=14, classifying all $3^{392}$ (≈1.075 ×$10^{187}$) states according to their energy values is a difficult work. The maximum density of states for L=14 is

(4) $$\begin{array}{ccc} \Omega (560) & = & 27221850979582122307103887345705430249291271754\\ \; & \; & 41733421466810315091391978178807681189467107781\\ \; & \; & 07857357109307134859979794274064012415824561064\\ \; & \; & 780628764327040290985201744055718050939294929, \end{array}$$

which is approximately $2.722\times 10^{185}$.

The simplest enumeration method for evaluating the exact integer values of Ω(E) is direct counting. Counting directly all $3^{N_{s}}$ states of the spin-one Ising model is possible for smaller lattices, such as $L_{x}=3$, 4, and 5. Because the algorithm of direct counting is simple, it is the best method for smaller lattices. However, for larger lattices, we need a more efficient enumeration method because the total number of states grows exponentially as $L_{x}$ increases. For some spin models, we can consider only two neighboring rows at a time. For two neighboring rows of the spin-one Ising model, the number of states is $3^{2L_{x}}$, very small compared to $3^{N_{s}}=3^{L_{x}L_{y}}$. Repeating the two-row $3^{2L_{x}}$ states $L_{y}-1$ times along the *y*-direction, we can evaluate efficiently the exact integer values of Ω(E) for larger lattices. This kind of enumeration is called the method of microcanonical transfer matrix [29], where the memory requirement is $2\left(\mathrm{rows}\right)\times 4\times P_{m}\times \left(E_{\max}+1\right)\times 3^{L_{x}}$ byte for the spin-one Ising model ($E_{\max}=2N_{b}$). Here, $P_{m}$ is the maximum size for storing very long integer numbers, such as Equation (4). For the spin-one Ising model, because of $\Omega \left(E\right)=\Omega \left(E_{\max}-E\right)$, we can reduce the memory requirement to $2\times 4\times P_{m}\times \left(N_{b}+1\right)\times 3^{L_{x}}$ byte. For example, the memory requirement is $2\times 4\times 3\times 66\times 3^{5}$ byte (≈385 kB) for L=5, whereas it is $2\times 4\times 20\times 561\times 3^{14}$ byte (≈429 GB) for L=14. In addition, the memory requirement is $2\times 4\times 23\times 646\times 3^{15}$ byte (≈1.706 TB) for L=15 whose exact enumeration is possible but not practical at the moment.

## 3. Partition Function Zeros in the Complex Temperature Plane of the Spin-Half Ising Model

The free energy, the specific heat, and the magnetization of the spin-half Ising model on honeycomb lattice have been exactly known in the absence of an external magnetic field [3,4]. However, the exact susceptibility of the spin-half Ising model in two dimensions has never been known in the absence of an external magnetic field. The Hamiltonian of the spin-half Ising model on the honeycomb lattice with $N_{s}$ sites and $N_{b}$ bonds is given by

(5) $$H=\frac{J}{2}\sum_{\langle i,j\rangle }(1-\sigma_{i}\sigma_{j}),$$

where $\sigma_{i}$ can take values −1 or +1 at each site *i*. If we introduce an energy index ($E=0,1,2,\ldots ,N_{b}$)

(6) $$E=\frac{1}{2}\sum_{\langle i,j\rangle }(1-\sigma_{i}\sigma_{j}),$$

the partition function of the spin-half Ising model (a sum over all $2^{N_{s}}$ spin configurations) can be expressed as

(7) $$Z\left(y\right)=\sum_{E=0}^{N_{b}}\Omega \left(E\right)y^{E}.$$

**Figure 2:**
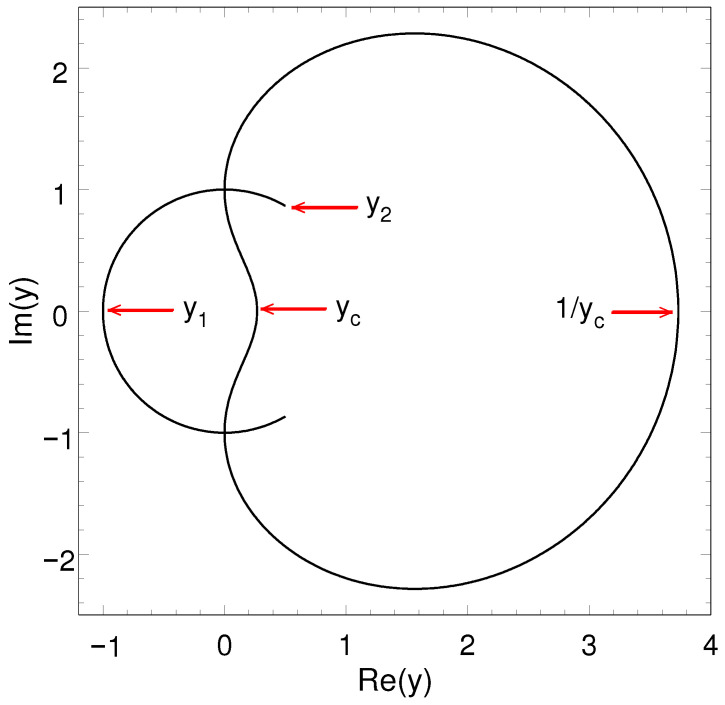
Distribution of the partition function zeros in the complex temperature ($y=e^{-\beta J}$) plane of the spin-half Ising model on the honeycomb lattice.

The distribution of the partition function zeros in the complex temperature plane for the spin-half Ising model on the honeycomb lattice has also been exactly known [27,28]. Figure 2 shows the exact distribution of the partition function zeros in the complex temperature ($y=e^{-\beta J}$) plane of the spin-half Ising model on the honeycomb lattice. As shown in the figure, the partition function zeros in the complex $y=e^{-\beta J}$ plane of the spin-half Ising model on the honeycomb lattice lie on the unit arc $y=e^{i\theta }$ ending at $y_{2}=\left(1\pm \sqrt{3}i\right)/2$ and the heart-like closed loop, crossing the positive real axis at the ferromagnetic critical point $y_{c}=2-\sqrt{3}\approx 0.267949$ and the antiferromagnetic critical point $1/y_{c}=2+\sqrt{3}\approx 3.732051$.

The properties of the partition function zeros in the complex temperature plane of the spin-half Ising model determine the behaviors of various thermodynamic functions, particularly their singular behaviors [28]. Consequently, the information on the properties of the complex-temperature singularities is invaluable in searching the closed-form expressions for the unknown thermodynamic functions such as the susceptibility of the Ising model in two dimensions. The partition function zeros of the spin-half Ising model on the honeycomb lattice show an interesting complex-temperature singularity $y_{1}=-1$ (on the unit arc) whose critical exponents are $\alpha_{1}=2$, $\beta_{1}=-1/4$, and $\gamma_{1}=2.4\left(3\right)$ (probably, 5/2) [28]. The values of $\alpha_{1}$ and $\beta_{1}$ are exactly obtained, and the value of $\gamma_{1}$ is approximately estimated from the low-temperature susceptibility series [28]. These values imply $\alpha_{1}+2\beta_{1}+\gamma_{1}=4$, different from the equality α+2β+γ=2 at the critical point $y_{c}$. There is another interesting complex-temperature singularity $y_{2}=\left(1\pm \sqrt{3}i\right)/2$, corresponding to the end point of the unit arc in Figure 2. The specific heat yields the critical exponent $\alpha_{2}=1$ at $y_{2}$ [28]. However, there is no exponent at $y_{2}$ from the spontaneous magnetization.

## 4. Partition Function Zeros in the Complex Temperature Plane of the Spin-One Ising Model

**Figure 3:**
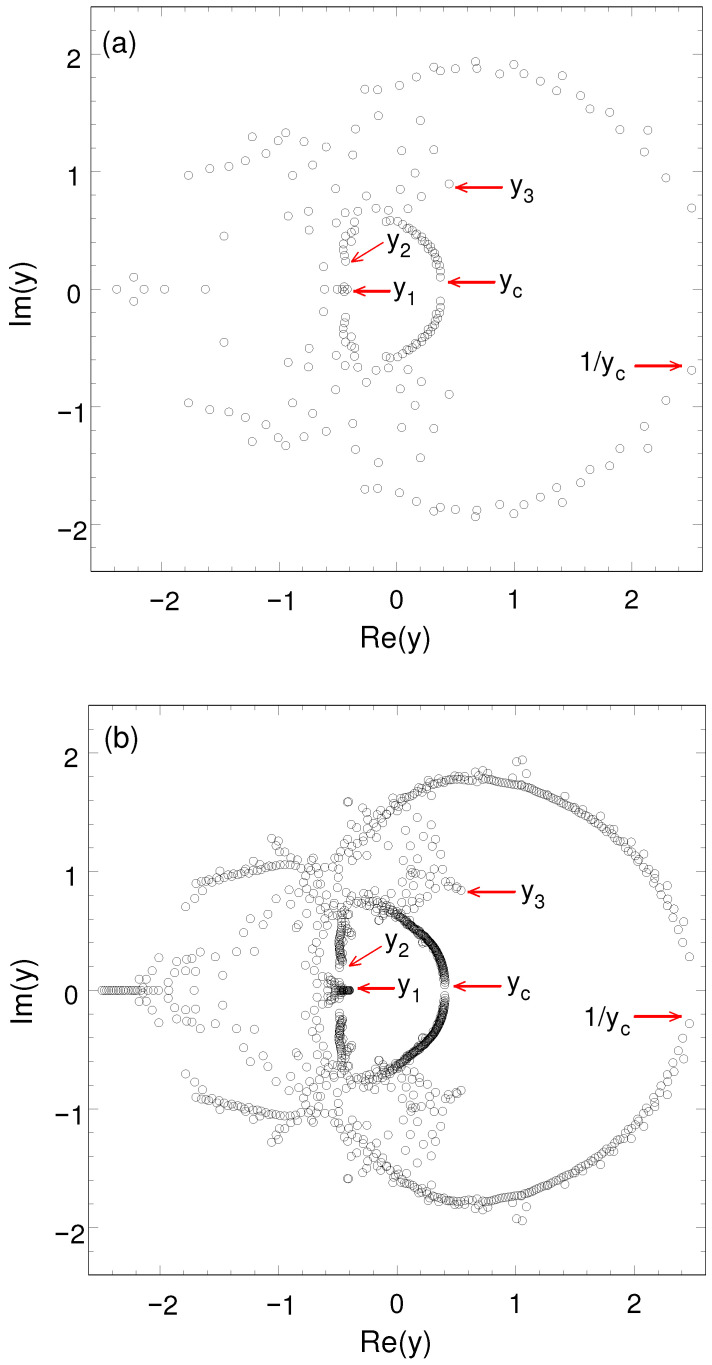
Partition function zeros in the complex $y=e^{-\beta J}$ plane of the spin-one Ising model on the L×2L honeycomb lattice for (**a**) L=6 and (**b**) L=14. The number of zeros is 192 (=$2N_{b}$) for L=6 and 1120 for L=14, respectively. It is expected that the zero $y_{c}\left(L\right)$ crosses the positive real axis at the ferromagnetic critical point in the limit L→∞. Similarly, the zero $1/y_{c}\left(L\right)$ crosses the positive real axis at the antiferromagnetic critical point. The partition function zeros $y_{1}$, $y_{2}$, and $y_{3}$ are possible candidates where the thermodynamic functions may show interesting behaviors.

The precise distributions of the partition function zeros $\left\{y_{k}\left(x\right)\right\}$ ($k=1,2,\ldots ,2N_{b}$) in the complex temperature ($y=e^{-\beta J}$) plane of the spin-one Ising model can be obtained from the exact partition functions on the L×2L honeycomb lattices:

(8) $$Z\left(y\right)=A\prod_{k=1}^{2N_{b}}(y-y_{k}),$$

where *A* is a constant. Figure 3 shows the partition function zeros in the complex $y=e^{-\beta J}$ plane of the spin-one Ising model on the L×2L honeycomb lattice for L=6 and L=14. The number of the partition function zeros is 192 (=$2N_{b}=6L^{2}-4L$) for L=6 and 1120 for L=14, respectively.

As shown in the figure, there is the partition function zero $y_{c}\left(L\right)$ closest to the positive real axis. We expect that $y_{c}\left(L\right)$ crosses the positive real axis, in the limit L→∞, at the ferromagnetic critical point $y_{c}$ whose exact value is not known. Similarly, the partition function zero $1/y_{c}\left(L\right)$ crosses the positive real axis at the antiferromagnetic critical point. Table 2 shows the partition function zero $y_{c}\left(L\right)$, closest to the positive real axis, of the spin-one Ising model on the L×2L honeycomb lattice for L= 5∼14. By using the Bulirsch–Stoer (BST) extrapolation method [36,37,38], we obtain the extrapolated value $y_{c}=0.42191\left(1\right)-0.0000003\left(57\right)i$ in the limit L→∞. The error estimates of the BST extrapolated values are conveniently measured as twice the difference between the (n−1, 1) and the (n−1, 2) approximants; they are not statistical. The BST extrapolated value for the ferromagnetic critical point is in agreement with $y_{c}=0.4217\left(10\right)$ estimated from the low-temperature series expansion [39]. The critical point $y_{c}^{\mathrm{hc}}=2-\sqrt{3}$ (≈0.267949) of the spin-half Ising model on the honeycomb lattice is connected to the critical point $y_{c}^{\mathrm{tr}}=1/\sqrt{3}$ (≈0.577350) on the triangular lattice by means of the star–triangle relation [3]:

(9) $$y_{c}^{\mathrm{tr}}=\frac{1-y_{c}^{\mathrm{hc}}}{1+y_{c}^{\mathrm{hc}}}.$$

It is an interesting question whether the star–triangle relation can be applied to the spin-one Ising model. By the star–triangle relation, the critical point $y_{c}^{\mathrm{hc}}=0.4217\left(10\right)$ of the spin-one Ising model on the honeycomb lattice gives the value of $y_{c}^{\mathrm{tr}}=0.4068\left(10\right)$. However, this value is in disagreement with $y_{c}^{\mathrm{tr}}=0.6875\left(15\right)$ estimated from the low-temperature series expansion [39].

The imaginary part Im[yc(L)] of the partition function zero $y_{c}\left(L\right)$ vanishes, in the limit L→∞, following the finite-size scaling [40]:

(10) Im[yc(L)]∼L−yt,

where $y_{t}$ is the thermal scaling exponent, determining the correlation-length critical exponent $\nu =1/y_{t}$ and the specific-heat critical exponent $\alpha =2-d/y_{t}$. From Equation (10), we can calculate the effective thermal scaling exponent

(11) yt(L)=−ln{Im[yc(L+1)]/Im[yc(L)]}ln[(L+1)/L]

for the spin-one Ising model on the L×2L honeycomb lattice. The third column of Table 2 shows the values of the effective thermal scaling exponent $y_{t}\left(L\right)$. The BST extrapolated value for the thermal scaling exponent of the spin-one Ising model on the honeycomb lattice is $y_{t}=1.0001\left(6\right)$, implying $y_{t}=1$.

Figure 3 shows the partition function zero $y_{1}$ on the real axis of the spin-one Ising model on the honeycomb lattice, somewhat similar to the complex-temperature singularity $y_{1}=-1$ on the unit arc of the spin-half Ising model. In the figure, the values of $y_{1}\left(L\right)$ of the spin-one Ising model are −0.41999055 for L=6 and −0.40200337 for L=14, respectively. Also, the partition function zeros $1/y_{1}\left(L\right)$ appear in the figure such as −2.38100598 for L=6 and −2.48754133 for L=14. Without loss of generality, we deal only with the partition function zeros on the unit disk |y|≤1. The BST extrapolated value for the partition function zero $y_{1}$ of the spin-one Ising model is $y_{1}=-0.398\left(6\right)$. Fox and Guttmann [39] obtained the singularity y=−0.3980(10), closer to the origin than the ferromagnetic critical point $y_{c}=0.4217\left(10\right)$, in the low-temperature series expansion for the spin-one Ising model on the honeycomb lattice. The BST extrapolated value for $y_{1}$ is in agreement with the location of the singularity in the low-temperature series expansion.

The critical exponents, $\alpha_{1}$, $\beta_{1}$, and $\gamma_{1}$, associated with the complex-temperature singularity $y_{1}$ can be defined in the usual way:

(12) $$C∼{\left(1-\frac{y}{y_{1}}\right)}^{-\alpha_{1}},$$

(13) $$m_{0}∼{\left(1-\frac{y}{y_{1}}\right)}^{\beta_{1}},$$

and

(14) $$\chi ∼{\left(1-\frac{y}{y_{1}}\right)}^{-\gamma_{1}},$$

where *C*, $m_{0}$, and χ are the specific heat, the spontaneous magnetization, and the susceptibility, respectively. To investigate the unknown properties of the critical exponent at the complex-temperature singularity $y_{1}$, we use the low-temperature series expansion for the spin-one Ising model on the honeycomb lattice [39]. We estimate the values of $\alpha_{1}$, $\beta_{1}$, and $\gamma_{1}$ by using Dlog Padé approximants [41] to the specific heat, the spontaneous magnetization, and the susceptibility. The [N/D] Padé approximant to a function f(y) is the quotient of two polynomials $P_{N}\left(y\right)$ and $Q_{D}\left(y\right)$ of degree *N* and *D*, respectively. Table 3 shows the values of $\beta_{1}$ and $y_{1}$ estimated from [N/D] Dlog Padé approximants to the spontaneous magnetization of the spin-one Ising model on the honeycomb lattice. The average values for $\beta_{1}$ and $y_{1}$ in the table are $\beta_{1}=-0.13\left(2\right)$ and $y_{1}=-0.398\left(4\right)$, respectively. Similarly, Dlog Padé approximants to the specific heat and the susceptibility yield $\alpha_{1}=1.15\left(51\right)$ and $y_{1}=-0.397\left(15\right)$ and $\gamma_{1}=1.21\left(53\right)$ and $y_{1}=-0.398\left(4\right)$, respectively. That is, the denominators of the unknown thermodynamic functions of the spin-one Ising model on the honeycomb lattice should include the terms proportional to

(15) $${\left(1+\frac{y}{|y_{1}|}\right)}^{\epsilon },$$

where ϵ is $\alpha_{1}$ for the specific heat, $-\beta_{1}$ for the spontaneous magnetization, and $\gamma_{1}$ for the susceptibility, respectively. For example, the divergence at $y_{1}$ is the inherent property of the unknown spontaneous magnetization. These values of the critical exponents at the complex-temperature singularity $y_{1}$ give $\alpha_{1}+2\beta_{1}+\gamma_{1}=2.1\left(7\right)$, probably satisfying the equality α+2β+γ=2 at the ferromagnetic critical point $y_{c}$. The estimated values of $\alpha_{1}=1.15\left(51\right)$, $\beta_{1}=-0.13\left(2\right)$, and $\gamma_{1}=1.21\left(53\right)$ for the spin-one Ising model on the honeycomb lattice are rough but different from $\alpha_{1}=2$, $\beta_{1}=-1/4$, and $\gamma_{1}=5/2$ for the spin-half Ising model. Rather, the values of the critical exponents $\alpha_{1}$, $\beta_{1}$, and $\gamma_{1}$ for the spin-one Ising model on the honeycomb lattice are similar to the conjectured values $\alpha_{e}=7/6$, $\beta_{e}=-1/6$, and $\gamma_{e}=7/6$ at the Fisher edge singularity of the *Q*-state Potts model in two dimensions [42]. The physical critical point of the *Q*-state Potts model disappears in a positive magnetic field, whereas the Fisher edge singularity appears in the complex temperature plane.

Figure 3 shows an interesting partition function zero $y_{2}$ of the spin-one Ising model on the honeycomb lattice whose BST estimated value is $y_{2}=-0.5\left(1\right)+0.2\left(1\right)i$. Table 4 shows the values of $\alpha_{2}$ and $y_{2}$ estimated from [N/D] Dlog Padé approximants to the specific heat of the spin-one Ising model on the honeycomb lattice. The average values for $\alpha_{2}$ and $y_{2}$ in the table are $\alpha_{2}=-0.81\left(31\right)$ and $y_{2}=-0.45\left(4\right)+0.25\left(5\right)i$, respectively. There is no exponent at $y_{2}$ from the spontaneous magnetization of the spin-one Ising model. This situation is similar to that of the spin-half Ising model at the complex-temperature singularity $y_{2}=\left(1+\sqrt{3}i\right)/2$.

In the figure, there is another interesting partition function zero $y_{3}$ of the spin-one Ising model on the honeycomb lattice, lying on the unit circle $y=e^{i\theta }$. The values of $y_{3}\left(L\right)$ and $\theta_{3}\left(L\right)$ of the spin-one Ising model are $y_{3}=0.44585237+0.89510651i$ and $\theta_{3}=1.10867004$ for L=6 and $y_{3}=0.53846967+0.84264489i$ and $\theta_{3}=1.00217637$ for L=14, respectively. The BST extrapolated values are $y_{3}=0.563\left(7\right)+0.826\left(2\right)i$ and $\theta_{3}=0.973\left(1\right)$. These values for the spin-one Ising model on the honeycomb lattice are not distant from the complex-temperature singularity $y_{2}=\left(1+\sqrt{3}i\right)/2$ and $\theta_{2}=\pi /3\approx 1.047$ for the spin-half Ising model. Unfortunately, the low-temperature series expansion for the spin-one Ising model on the honeycomb lattice gives no information on the properties of $y_{3}$. Instead, we assume a plausible finite-size scaling

(16) $$\theta_{3}\left(L\right)-\theta_{3}∼L^{-y_{t}^{\left(3\right)}},$$

where $y_{t}^{\left(3\right)}$ is the thermal scaling exponent at $y_{3}$. Then, imitating Equation (11), we obtain the BST extrapolated value of $y_{t}^{\left(3\right)}=1.6\left(3\right)$. Again assuming $\alpha_{3}=2-d/y_{t}^{\left(3\right)}$, we reach the value of $\alpha_{3}=0.7\left(3\right)$. Interestingly, the value of $\alpha_{3}=0.7\left(3\right)$ at $y_{3}$ for the spin-one Ising model on the honeycomb lattice is not far from $\alpha_{2}=1$ at the complex-temperature singularity $y_{2}$ for the spin-half Ising model.

## 5. Conclusions

The exact integer values for the density of states Ω(E) of the spin-one Ising model on the L×2L honeycomb lattice with $N_{s}=2L^{2}$ sites and $N_{b}=3L^{2}-2L$ bonds ($E=0,1,2,\ldots ,2N_{b}$) are enumerated up to L=14. Therefore, the exact partition function Z(y) of the spin-one Ising model can be constructed as a polynomial in the low-temperature variable $y=e^{-\beta J}$. Then, the precise distributions of the partition function zeros $\left\{y_{k}\left(x\right)\right\}$ ($k=1,2,\ldots ,2N_{b}$) in the complex temperature ($y=e^{-\beta J}$) plane of the spin-one Ising model are evaluated from the exact partition functions on the L×2L honeycomb lattices. The various properties of the spin-one Ising model on the honeycomb lattice are investigated, based on its partition function zeros in the complex *y* plane.

We obtain the ferromagnetic critical point $y_{c}=0.42191\left(1\right)$ of the spin-one Ising model on the honeycomb lattice, in agreement with the previous estimate $y_{c}=0.4217\left(10\right)$ by the low-temperature series expansion. The value of $1/y_{c}$ corresponds to the antiferromagnetic critical point. At $y_{c}$, we obtain the thermal scaling exponent $y_{t}=1$, implying the critical exponents ν=1 and α=0, as expected. Also, we obtain the complex-temperature singularity $y_{1}=-0.398\left(6\right)$ where the specific heat, the spontaneous magnetization, and the susceptibility of the spin-one Ising model on the honeycomb lattice diverge with the critical exponents $\alpha_{1}=1.15\left(51\right)$, $\beta_{1}=-0.13\left(2\right)$, and $\gamma_{1}=1.21\left(53\right)$, probably satisfying the equality α+2β+γ=2. Those are somewhat different from the properties of the complex-temperature singularity $y_{1}=-1$ of the spin-half Ising model on the honeycomb lattice with $\alpha_{1}=2$, $\beta_{1}=-1/4$, and $\gamma_{1}=5/2$, implying α+2β+γ=4. In addition, for the spin-one Ising model on the honeycomb lattice, we notice other interesting partition function zeros $y_{2}=-0.5\left(1\right)+0.2\left(1\right)i$ with $\alpha_{2}=-0.81\left(31\right)$ and $y_{3}=0.563\left(7\right)+0.826\left(2\right)i$ (on the unit circle) with $\alpha_{3}=0.7\left(3\right)$.

## Figures and Tables

**Table 1:** Exact integer values for the density of states Ω(E) of the spin-one Ising model on the L×2L honeycomb lattice for L=5 with $N_{s}=2L^{2}=50$ sites and $N_{b}=3L^{2}-2L=65$ bonds. In the table, the values for $E=N_{b}+1∼2N_{b}$ are omitted because of $\Omega \left(E\right)=\Omega \left(2N_{b}-E\right)$.

*E*	Ω(E)	*E*	Ω(E)	*E*	Ω(E)
0	2	1	4	2	38
3	152	4	630	5	2526
6	9264	7	33378	8	113300
9	381118	10	1224262	11	3884972
12	11981892	13	36388260	14	108394822
15	318094328	16	920144104	17	2624353476
18	7395805566	19	20573186210	20	56589898500
21	153772977216	22	413207639432	23	1097272921008
24	2880626587654	25	7473246385172	26	19158682797178
27	48521300452188	28	121364677125906	29	299726874362580
30	730570907926620	31	1756955371483898	32	4166982072260800
33	9742508416092690	34	22443377287488990	35	50917265054383526
36	113700039907873148	37	249765605402710676	38	539407597917215078
39	1144563479032772032	40	2384574997304753550	41	4874452089298938606
42	9769364659249329556	43	19182206092710453896	44	36870271985537794496
45	69316760199842711544	46	127353857752153004412	47	228461510023823141290
48	399804993237498547262	49	681893860793446171682	50	1132438909118233396464
51	1829520165423363819674	52	2872653179562151335310	53	4379855651227359864260
54	6478691413028990374560	55	9289740540936939336702	56	12902354441497779407244
57	17344825759457153166140	58	22553947224781995217912	59	28351418677876309288560
60	34435636989562342178034	61	40395993011520198167262	62	45752566826399621994736
63	50017727963173270718994	64	52769344481784076002320	65	53720458126972059836125

**Table 2:** Partition function zero $y_{c}\left(L\right)$, closest to the positive real axis, of the spin-one Ising model on the L×2L honeycomb lattice. Here, $y_{t}\left(L\right)$ is the effective thermal scaling exponent.

*L*	$y_{c}\left(L\right)$	$y_{t}\left(L\right)$
5	0.35926358+0.12034975i	0.91911736
6	0.37054358+0.10178138i	0.92905684
7	0.37835423+0.08820049i	0.93668382
8	0.38408991+0.07783069i	0.94277533
9	0.38848380+0.06965071i	0.94777449
10	0.39195906+0.06303151i	0.95195908
11	0.39477737+0.05756435i	0.95551733
12	0.39710941+0.05297195i	0.95858187
13	0.39907132+0.04905956i	0.96124984
14	0.40074492+0.04568631i	

**Table 3:** Values of $\beta_{1}$ and $y_{1}$ estimated from [N/D] Dlog Padé approximants to the spontaneous magnetization of the spin-one Ising model on the honeycomb lattice.

[N/D]	$\beta_{1}$	$y_{1}$
[6/6]	−0.111910	−0.394053
[7/6]	−0.125741	−0.397768
[6/7]	−0.132779	−0.399290
[8/7]	−0.132957	−0.399430
[8/8]	−0.128397	−0.398484
[7/8]	−0.139954	−0.400749
[9/8]	−0.116744	−0.396256
[9/9]	−0.128332	−0.398470
[8/9]	−0.123773	−0.397541
[10/9]	−0.132908	−0.399417
[9/10]	−0.140364	−0.400816
[11/10]	−0.125544	−0.397715
[11/11]	−0.110571	−0.393567
[10/11]	−0.133755	−0.399487

**Table 4:** Values of $\alpha_{2}$ and $y_{2}$ estimated from [N/D] Dlog Padé approximants to the specific heat of the spin-one Ising model on the honeycomb lattice.

[N/D]	$\alpha_{2}$	$y_{2}$
[7/7]	−0.783553	−0.450863+0.236109i
[6/7]	−0.701654	−0.435259+0.261757i
[8/7]	−1.150912	−0.487611+0.211834i
[8/8]	−0.794063	−0.453029+0.232879i
[9/9]	−0.778174	−0.449728+0.237900i
[8/9]	−0.778609	−0.448433+0.285271i
[9/10]	−0.701783	−0.435137+0.262727i

## Data Availability

The original contributions presented in this study are included in the article. Further inquiries can be directed to the corresponding author.
